# Stressors and Life Satisfaction in Older Adults: The Moderating Role of Self-Efficacy

**DOI:** 10.1177/00914150251317441

**Published:** 2025-02-12

**Authors:** Minh Ngoc Pham, Sunil Bhar

**Affiliations:** Department of Psychological Sciences, 3783 Swinburne University of Technology, Victoria, Australia

**Keywords:** life events, hassles, self-efficacy, life satisfaction

## Abstract

**Objectives:** Understanding the factors protecting life satisfaction in older adults despite stressors is central to late-life wellbeing. This study examined whether self-efficacy moderated the relationships between negative life events, hassles, and life satisfaction in older adults. **Methods:** The sample comprised 176 older adults aged 60 or above. Two moderation models were tested, controlling for gender, socioeconomic status and health status. **Results:** The negative relationship between adverse life event and life satisfaction was stronger for individuals with lower self-efficacy. There was no evidence that self-efficacy moderated the relationship between hassles and life satisfaction. **Discussion:** The study is the first to provide empirical evidence for the role of self-efficacy in buffering the association between negative life events and lower life satisfaction. Strategies to increase self-efficacy can be incorporated in interventions and policies to enhance resilience in this growing population.

Old age is typically characterized as a time of increasing vulnerability to stressors, such as loss and grief, role changes, major illness, disability, and increasing dependency (Hannaford et al., 2018). Surprisingly, research has generally shown that older people reported higher levels of life satisfaction compared to younger groups (Blanchflower & Oswald, 2004; Park et al., 2020). Given this apparent resilience, it is important to understand the factors that enable older individuals to maintain high levels of life satisfaction despite adverse stressors in late life.

There is a paucity of research on the relationship between different types of stressors and wellbeing in older adults. Stressors refer to the objective environmental demands exerted on the individuals (Cohen et al., 2016). Two types of stressors have been commonly studied in the context of developmental stages: negative life events and hassles (Aldwin, 2011). Negative life events refer to major, time-discrete situations that occur to individuals such as bereavement or divorce. Daily stressors, or hassles, are minor and relatively short-duration problems that characterize individuals’ everyday transactions with the environment, such as misplacing items or arguing with one's children (Kanner et al., 1981). Research has shown that different types of stressors can have distinct and independent contributions to the variance in psychological outcomes. For example, in a meta-analysis of 25 studies between 1980 and 1998 on stressors and depression in older adults, Kraaij et al. (2002) found that the combined effect size of negative life events on depression was weaker than hassles on depression. Given the multifaceted nature of stressors in late life, a more realistic and comprehensive assessment of stressors needs to include both negative life events and hassles.

Life satisfaction is defined as an individual's global evaluation of their life (Pavot et al., 1991). Life satisfaction has garnered increasing attention among gerontology researchers, having established its association with better physical health as well as positive psychosocial outcomes in older adults (Kim et al., 2021; Sowa et al., 2016; Von Humboldt & Leal, 2017). However, research on the relationship between late-life stressors and life satisfaction in older adults remains limited. The majority of past research focused on examining stressors as risk factor for depression (Kraaij et al., 2002). Given the definition of health by World Health Organization (2006) as “a state of complete physical, mental, and social well-being and not merely the absence of disease or infirmity,” it is equally important to understand how stressors are associated with life satisfaction in late life. Understanding this association is also relevant to explain older individuals’ resilience, their ability to maintain healthy psychological functioning in spite of exposure to stressors (Cosco et al., 2017; Kiosses & Sachs-Ericsson, 2020).

The question of for whom life satisfaction is most adversely impacted by stressors remains to be explored. Self-efficacy, the foundation of human agency in social cognitive theory, is suggested to be one of the most promising constructs that can buffer the impact of stressors on individuals’ well-being (Bandura et al., 1999; Jerusalem & Mittag, 1995). Self-efficacy refers to an individual's confidence in their capability to organize and perform a course of action to attain a desired outcome (Bandura, 1977). There has been some empirical evidence for the stress-buffering role of self-efficacy across different populations. A number of studies in the field of occupational psychology have demonstrated self-efficacy's moderating role in the relationships between stressors and mental health as well as physical health (Peng et al., 2015; Siu et al., 2007). There has been some, but less research on the extent to which self-efficacy moderates the relationships between stressors and life satisfaction. For example, a study in adolescents found self-efficacy to moderate the relationships between interpersonal and school-related stressors and life satisfaction (Moksnes et al., 2019).

## Stressors and Life Satisfaction

Research into the relationship between negative life events and life satisfaction specifically in older adults has yielded inconsistent findings. In a longitudinal study, increased exposure to interpersonal stressful life events was associated with lower life satisfaction (Lee et al., 2022). On the contrary, some studies have reported no significant association between negative life events and life satisfaction (Hillerås et al., 2001). Additionally, some studies have shown that the relationship between negative life events and life satisfaction differs across populations. For example, one study found that the number of stressful life events was significantly associated with reduced life satisfaction only in the 50–64 age group, but not in the older group (Hannaford et al., 2018). Another study found that role loss events, such as retirement, divorce, and widow predicted low life satisfaction only for men, but not for women (Elwell & Maltbie-Crannell, 1981). The reasons for such variations in the relationship between negative life events and life satisfaction remain to be investigated.

In the context of stressful life events, there are three possible mechanisms via which an elevated sense of self-efficacy may enable individuals to effectively mitigate the adverse impact of stressors on their functioning and wellbeing (Benight & Bandura, 2004; Jerusalem & Mittag, 1995). First, in primary appraisal of stressors, highly self-efficacious individuals may perceive the environmental demands more as challenges than as threats. Second, in evaluating and employing their coping options, they remain motivated to manage their personal functioning and resources to cope with the stressors. Third, a robust sense of self-efficacy enables individuals to persevere in recovery efforts following their experience of traumatizing events (Benight & Bandura, 2004). As a result, individuals with high self-efficacy purportedly experience lower stress and anxiety, maintain positive outlook and have fewer health complaints when in stressful situations, compared to individuals with low self-efficacy (Benight & Bandura, 2004; Jerusalem & Mittag, 1995).

Despite the purported benefits of self-efficacy on outcomes, research on the stress-buffering effects of self-efficacy in older populations is lacking. In one qualitative study, self-efficacy emerged as a central theme in older adults’ narratives of their coping behaviors against both negative life events such as losses and major illness as well as hassles such as social disputes and misplacing things (Schneller & Vandsburger, 2008). Another study among Chinese middle-aged and older women suggested that self-efficacy and financial resources can help maintain perceived quality of life among individuals facing adverse life events (Leung & Liu, 2021). Conversely, a study conducted among spousal dementia caregivers found no evidence that self-efficacy moderated the relationship between caregiving hassles and perceived stress and caregiver burden (Pertl et al., 2017). None of these studies investigated the relationship between life satisfaction, self-efficacy and stressors, and there is not enough evidence to compare the effectiveness of self-efficacy in mitigating the impact of different stressor types on life satisfaction in older adults.

In summary, the relationships between life satisfaction and different types of stressors have rarely been examined together. It is unclear which psychological factors help maintain life satisfaction in the presence of such stressors among older individuals. The aim of the present study was to investigate whether individual differences in self-efficacy moderated the relationships between two types of stressors and life satisfaction in older adults. Two hypotheses were proposed. We hypothesized that higher self-efficacy would lessen the negative impacts of higher number of negative life events on life satisfaction. We also hypothesized that higher self-efficacy would lessen the negative impacts of higher number of hassles on life satisfaction. These hypotheses were investigated while controlling for gender, socioeconomic status, and health status, all of which have commonly been found to be related to both stressors and life satisfaction (Baum et al., 1999; Elwell & Maltbie-Crannell, 1981; Luhmann & Eid, 2009; Park et al., 2020).

## Methods

### Research Design

The study used a quantitative, cross-sectional design. Data were collected via a community survey of older adults living in Australia. The survey was completed online (93%) or by in-person interview (7%). Ethics approval for the study was granted by the Swinburne University Human Research Ethics Committee, reference number 20237112–15682.

### Data Collection

Data were collected using the survey management tool Qualtrics from June to August 2023. The study involved completing self-report measures of life satisfaction, general self-efficacy, negative life events, daily hassles, and sociodemographic information. The order of measures was consistent in both data collection modes. Details of the measures are as following:

#### Life Satisfaction

Satisfaction with Life Scale (SWLS; Diener et al., 1985) is a five-item instrument that assesses an individual's global evaluation of satisfaction with one's life. Participants rated their agreement with statements such as “If I could live my life over, I would change almost nothing” on a 7-point Likert scale, ranging from *strongly disagree* (1) to *strongly agree* (7). Item scores were added to produce a total score ranging from 5 to 35, with higher scores suggesting higher life satisfaction. The SWLS has shown strong internal reliability (Cronbach's α = .82−.87) and construct validity: correlations were as expected between the SWLS and measures of depression (*r *= −.72), negative affect (*r *= −.48), and positive affect (*r *= .44; Pavot & Diener, 1993; Von Humboldt & Leal, 2017). Internal reliability of the SWLS in the current study was excellent (Cronbach's α = .86).

#### General Self-Efficacy

The Generalized Self-Efficacy (GSE; Schwarzer & Jerusalem, 1995) is a 10-item scale that measures one's beliefs about one's ability to cope with challenging situations. Participants rate statements such as “I can usually handle whatever comes my way” on a 4-point scale from *not at all true* (1) to *exactly true* (4). Item scores were added to produce a GSE total score ranging from 10 to 40, with higher scores indicating higher levels of self-efficacy. The GSE demonstrated high reliability (Cronbach's α = .75−.91) and construct validity: the GSE correlated in the expected direction with measures of depression (*r *= −.33 to −.46), and optimism (*r *= .52 to .60; Scholz et al., 2002). In the current study, the GSE had excellent internal reliability (Cronbach's α = .90).

#### Negative Life Events

Recent negative life events were measured using the 12-item List of Threatening Experiences (LTE; Brugha & Cragg, 1990). The inventory includes 12 major life events, which are typically perceived as highly stressful, such as experiencing a major illness or the loss of a significant other. Participants indicated if they have/have not experienced each negative life event in “the past 6 months.” Occurrences of events were summed to produce a total score ranging from 0 to 12, and higher scores indicate having experienced more negative life events in the past 6 months. LTE has shown strong concurrent validity based on independent rating (sensitivity at 0.89 and specificity at 0.74 for events in last 6 months; Brugha & Cragg, 1990). The Cronbach's α for the LTE in the current sample was .58. However, it is important to note that internal consistency is not a required criterion for measures of life events (Cleary, 1981).

#### Daily Hassles

Hassles were assessed using a 25-item revised hassles index by Holahan and Holahan (1987). This revised index was based on the original 117-item Hassles Scale by Kanner et al. (1981). The selected 25 items were the most frequently reported hassles by middle-aged and older adults, such as “misplaced or lost things” or “problems of ageing parents.” In the original Hassles Scale by Kanner et al. (1981), participants were instructed to rate each hassle that they experienced in severity with score from 1 = “*somewhat severe*” to 3 = “*extremely severe*,” which later were summed into a total hassle severity score for analysis. In the revised index, Holahan and Holahan (1987) modified the instruction to replace “severity” rating with a dichotomous rating of yes/no to indicate whether each hassle has occurred in the past month. The number of hassles reported was summed to create a total score for each participant ranging from 0 to 25, with higher scores indicating a greater number of hassles over the past month. In the current study, the revised hassle index showed good internal consistency (Cronbach's α = .77), though this is not a required criterion for measures of hassles.

#### Sociodemographic Covariates

Sociodemographic variables were measured with a purpose-built demographic form. The form comprised questions asking participants’ gender, annual gross household income, income satisfaction, and subjective health status. Annual gross household income was categorized with reference to Australian Bureau of Statistics 2019–2020 quintile brackets (Australian Bureau of Statistics, 2020). Income satisfaction was evaluated by participants’ responses to “Which one of these phrases comes closest to your own feelings about your household's income these days?” (Park et al., 2020). Subjective health status was assessed with the question: “Do you have any health problems that prevent you from doing any of the things people your age normally can do?” (Park et al., 2020). Participants responded yes or no to this question.

### Participants

The sample comprised of 176 older adults (M_age _= 73.10, SD_age _= 8.66, range = 60 to 97 years).

Participants were eligible for the study if they were at least 60 years old, were living in Australia and could complete the questionnaire independently in English. Exclusion criteria were the presence of cognitive or sensory difficulties that could interfere with study task completion.

The study was promoted through multiple methods to recruit participants: information about the study was distributed via (i) the researchers’ social media (e.g., Facebook, LinkedIn), (ii) word-of-mouth, researchers’ personal email networks; (iii) emailing professional mailing lists (e.g., the Swinburne Wellbeing Clinic for Older Adults, JoinUs.org.au), (iv) online announcements on the Wellbeing Clinic for Older Adults website and health professional websites—such as the Australian Psychological Society and (v) displaying posters at community centers, public libraries, churches, nursing homes and senior clubs. No compensation was offered for participation in the study.

### Data Analysis

The minimum sample size was calculated with G*Power. Using F tests, Linear multiple regression: Fixed model, R^2^ increase, assuming effect size *f^2 ^*= .05, alpha level = .05 and power = .80, testing the interaction effect with three predictor variables, the required sample size needed to be at least 160. The questionnaires were completed by 186 eligible individuals. Nine participants were excluded from the analyses because their responses had more than 30% missing data in each of the main variables. One case of multivariate outlier was identified with Mahalanobis distance value equaled 29.98 and exceeded the Chi-square critical value of 16.27 with three degree of freedom (Tabachnick & Fidell, 2018), and was removed from the main analyses. The final sample of 176 participants rendered the study sufficiently powered for its primary analysis.

Moderation analyses were conducted using Model 1 in PROCESS macro version 4.3 in SPSS (Hayes, 2017). Two moderation models were tested with negative life events and hassles as the predictors, self-efficacy as the moderator and life satisfaction as the outcome, controlling for gender, annual gross household income, income satisfaction, and health status. Moderation analyses were performed with the number of bootstrap samples set at 5,000. Statistical significance was set at *p *< .05 level and 95% confidence interval. The predictor variables were centered for this analysis.

## Results

### Demographic Characteristics

Data from a total of 176 participants were included in the final analysis. The average age of the sample was 73.1 (*SD* = 8.66) with a range from 60 to 97 years. The sample had a majority of female (74.4%). Almost half of the sample had completed a postgraduate degree (47.7%) and the majority reported living comfortably on present income (69.9%). Detailed demographics of the sample are shown in Table 1.

**Table 1.** Demographic Characteristics of the Sample (*n* = 176).

Variables	Total (%)
Gender	
Male	43 (24.4)
Female	131 (74.4)
Missing	2 (1.2)
Born in Australia	130 (73.9)
Living environment	
Community-dwelling	164 (93.2)
Residential-care	12 (6.8)
Relationship status	
Married or in a de-facto relationship	108 (61.4)
Widowed, divorced, or separated	51 (29)
Never married	15 (8.5)
Highest level of education completed	
Postgraduate university	84 (47.7)
Undergraduate university	32 (18.2)
Year 12	20 (11.4)
Gross household income (annual)	
No income	7 (4)
Less than $25,000 (poorest 20%)	15 (8.5)
$25,001–$56,200 (second 20%)	36 (20.5)
$56,201–$93,100 (middle 20%)	33 (18.8)
$93,101–$143,200 (fourth 20%)	28 (15.9)
More than $143,200 (richest 20%)	22 (12.5)
Income satisfaction	
Finding it very difficult and difficult on present income	8 (4.5)
Getting by on present income	40 (22.7)
Living comfortably on present income	123 (69.9)
Work status	
Employed	50 (28.5)
Retired	120 (68.2)
Had health problems	36 (20.5)

### Descriptive Statistics

Table 2 shows descriptive statistics of the main variables in this sample (*n* = 176). Independent sample *t*-tests showed that there were no significant differences in the means of life satisfaction, self-efficacy, negative life events, and hassles between older adults who completed the online questionnaire and those who were interviewed.

**Table 2.** Descriptive Statistics of Main Variables for Participants.

Variables	Range	Total *M (SD)*	Questionnaire *M (SD)*	Interview *M (SD)*	*df*	*T*	*p*
Life satisfaction	7–35	26.01 (6.20)	26.01 (6.15)	26.08 (7.14)	174	−.04	.97
Self-efficacy	19–40	32.02 (4.37)	32.07 (4.22)	31.42 (6.22)	11.76	.36	.73
Life events	0–8	1.60 (1.61)	1.59 (1.62)	1.76 (1.54)	174	−.34	.73
Hassles	0–17	4.62 (3.63)	4.70 (3.69)	3.43 (2.55)	174	1.17	.24

### Correlational Analyses

Correlations between study variables are presented in Table 3. Negative life events and hassles were significantly negatively associated with life satisfaction. Life satisfaction was significantly positively correlated with self-efficacy, annual gross household income, income satisfaction, and health status. Number of negative life events was significantly positively associated with number of hassles. Hassles was significantly negatively correlated with gender indicating that women reported more hassles than men.

**Table 3.** Correlations Among Study Variables.

Variables	1	2	3	4	5	6	7	8
1. Life satisfaction	–	.36***	−.16*	−.48***	−.03	.19*	.20**	.15*
2. Self-efficacy		–	−.01	−.13	−.02	.11	.12	.05
3. Negative life events			–	.30***	−.03	−.04	−.07	.02
4. Hassles				–	.19*	−.11	−.12	.03
5. Gender					–	−.12	.14	.06
6. Household income						–	.07	−.00
7. Income satisfaction							–	.18*
8. Health status								–

*p < .05. **p < .01. ***p < .001.

The associations between self-efficacy and negative life events, and between self-efficacy and hassles were nonsignificant.

### Moderation Effect of Self-Efficacy

#### Negative Life Events × Self-Efficacy

**Figure 1.**
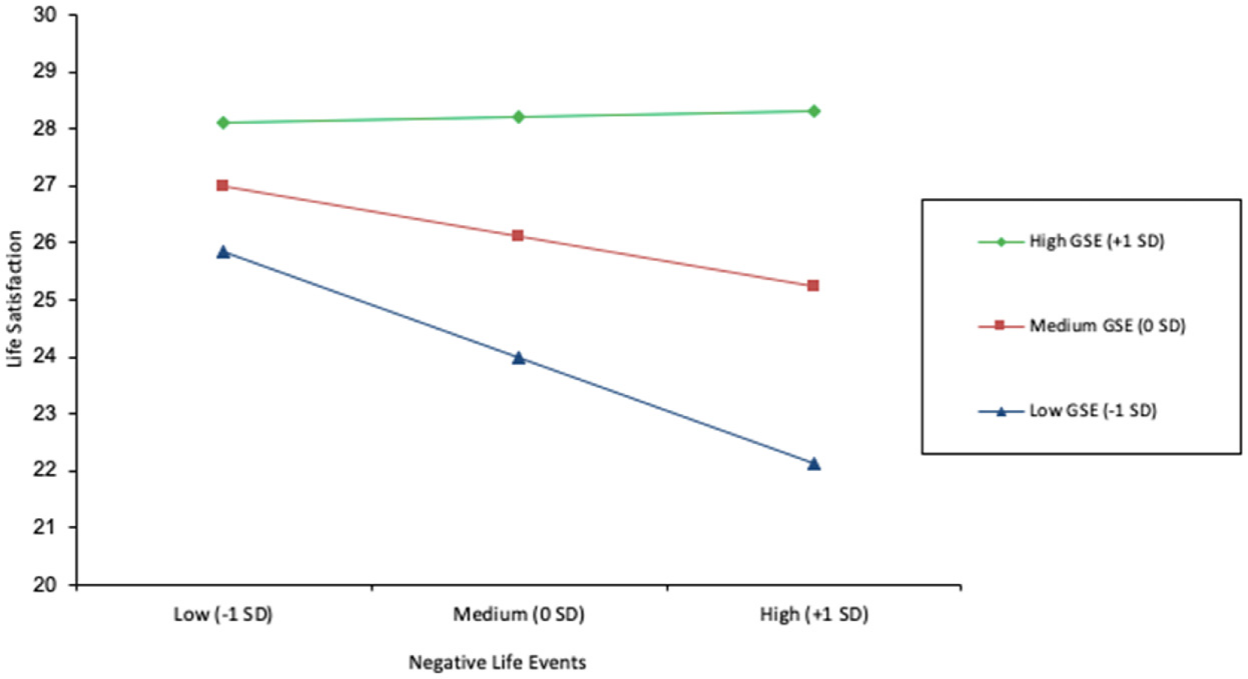
Interaction between negative life events and general self-efficacy in predicting life satisfaction.

The results of the Regression Model 1 (negative life events) are summarized in Table 4 and Figure 1. The regression model was significant R = .49, F (7, 166) = 7.45, *p *< .001 and collectively the predictors accounted for 24% of the variance in participants’ life satisfaction. Lower life satisfaction was associated with higher number of negative life events (B = −.55, *p *< .05) and lower self-efficacy (B = .48, *p *< .001). Additionally, there was a significant interaction effect between negative life events and self-efficacy (B = .14, *p *< .05) in predicting life satisfaction, which explained an additional 2% of the variance in life satisfaction, *F*(1,166) = 4.63, *p *< .05.

**Table 4.** Moderating Effect of General Self-Efficacy on the Relationship Between Negative Life Events and Life Satisfaction.

Variables	B	SE	*p*	R^2^	ΔR^2^
				.24***	.02*
Negative life events (NLE)	−.55*	.26	.03		
General self-efficacy (GSE)	.48***	.10	<.001		
NLE x GSE	.14*	.07	.03		
Gender	−.47	.98	.63		
Household income	.38	.20	.06		
Income satisfaction	.04	.02	.12		
Health status	.05	.03	.06		

*p < .05. ***p < .001.

Simple slopes tests showed that negative life events significantly and negatively predicted life satisfaction when individuals’ self-efficacy was low (−1 SD) (B = −1.16, *p *< .01) and medium (0 SD) (B = −.55, *p *< .05). In contrast, there was no significant association between negative life events and life satisfaction among individuals with high self-efficacy (+1 SD) (B = .06, *p *= .87).

### Hassles × Self-Efficacy

The results for Model 2 (Hassles) are summarized in Table 5. The regression model was significant R = .35, *F*(7, 166) = 13.02, *p *< .001 and the predictors accounted for 60% of the variance in participants’ life satisfaction. Lower life satisfaction was associated with higher number of hassles (B = −.71, *p *< .001) and lower self-efficacy (B = .40, *p *< .001). Interaction between hassles and self-efficacy on life satisfaction was nonsignificant; number of hassles significantly predicted life satisfaction, regardless of individuals’ level of self-efficacy.

**Table 5.** Moderating Effect of General Self-Efficacy on the Relationship Between Hassles and Life Satisfaction.

Variable	B	SE	*p*	R^2^	ΔR^2^
				.35***	.00
Hassles	−.71***	.11	<.001		
General self-efficacy (GSE)	.40***	.09	<.001		
Hassles x GSE	−.00	.02	.72		
Gender	.74	.92	.42		
Household income	.33	.19	.08		
Income satisfaction	.03	.02	.21		
Health status	.05*	.03	.04		

*p < .05. ***p < .001.

## Discussion

The present study investigated whether general self-efficacy moderated the extent to which negative life events and hassles were associated with life satisfaction in older adults. The first hypothesis was supported; general self-efficacy moderated the relationship between negative life events and life satisfaction, controlling for gender, socioeconomic and health status. The second hypothesis, however, was not supported; there was no evidence that general self-efficacy moderated the relationship between hassles and life satisfaction in older adults.

The findings provided evidence of the stress-buffering role of general self-efficacy in the relationship between negative life events and life satisfaction in older adults. In this study, older individuals with high self-efficacy appeared less susceptible to the impact of higher number of negative life events and exhibited more consistent levels of life satisfaction in comparison to those with lower self-efficacy. This finding provides, for the first time, empirical support for the notion that self-efficacy serves as a stress-buffer for older adults’ life satisfaction in the face of negative life events (Bandura et al., 1999; Benight & Bandura, 2004; Jerusalem & Mittag, 1995). The results corroborate and expand upon previous research (Leung & Liu, 2021; Qin et al., 2020; Schneller & Vandsburger, 2008), highlighting that general self-efficacy plays not only a predictive role in maintaining well-being but also a protective role in the context of distressing events in late life.

In contrast, the study did not find support for the role of self-efficacy in mitigating the negative association between hassles and life satisfaction in older adults. Higher number of hassles was significantly associated with individuals’ lower life satisfaction, irrespective of their levels of self-efficacy. This is not the first study to report the absence of a significant interaction effect between self-efficacy and hassles in predicting psychological outcomes in older populations. Previously, Pertl et al. (2017) found that self-efficacy did not moderate the relationship between caregiving hassles and the levels of perceived stress and caregiver burden among elderly spousal dementia caregivers.

The study offers a novel contribution in demonstrating that whether self-efficacy moderates the relationships between stressors and life satisfaction in older adults varies depending on the types of stressors—negative life events versus hassles. One possible explanation for the difference in findings is with reference to the distinction in the time duration of negative life events versus hassles. Life events are time-discrete occurrences that have already taken place in the past and may mark a prolonged adaptive process thereafter. In contrast, hassles, which encompass individuals’ everyday transaction with the environment, are more likely to represent immediate and ongoing stressors. The findings, which indicated that general self-efficacy buffered the adverse impacts of past life events but not of immediate hassles on life satisfaction, provide support for the notion that general self-efficacy may play a significant role in the latter stage of stress coping, particularly in the recovery process following past events. This is relevant in the context of recovery from traumatic events (Benight & Bandura, 2004). For example, in a study of recent widows, a strong sense of self-efficacy was found to help individuals effectively manage the bereavement process and maintain their psychological well-being (Benight et al., 2001).

The overarching theme that general self-efficacy protects life satisfaction against the adverse effect of negative life events suggest potential target for clinical intervention. General self-efficacy is a potentially modifiable resource (FitzGerald et al., 2022), and can be developed through therapeutic experiences such as mastery enactment, social modeling and correcting cognitive distortions (Benight & Bandura, 2004). Strengthening self-efficacy can be the target for therapy, support groups and services aiming to restore wellbeing among older individuals recovering from negative life events.

## Limitations and Future Directions

The study used a cross-sectional design, which can only indicate associations but cannot infer causal relationships among the variables. A longitudinal design, in which participants from low versus high self-efficacy groups report measures of stressors and life satisfaction over time, can clarify the direction of the interrelationships between these variables. The sample is homogeneous in terms of socioeconomic status—with nearly half of the participants having completed a postgraduate education and the majority reported high satisfaction with their income, hence limiting the generalizability of the findings. Future research can extend recruitment and data collection methods to enhance the participation of more socioeconomically diverse samples.

Another limitation pertains to the discrepant time frames in the stressors’ measurements, where participants reported negative life events in the last 6 months and hassles in the last one month. Future studies could measure when participants experienced specific stressors and account for this variation to more accurately evaluate the impact of stressors on life satisfaction and their interactions with self-efficacy. Additionally, since the interaction effect only explained an extra 2% of the variance in life satisfaction, caution needs to be taken in emphasizing the stress-buffering role of self-efficacy.

With regards to the measure of self-efficacy, past research indicated that domain-specific self-efficacy measures typically had better sensitivity in predicting changes in functioning compared to general self-efficacy (Bandura, 1989). Our findings suggest that general self-efficacy may facilitate the recovery process after negative life events. However, the stress-buffering effect of domain-specific self-efficacy and its potentially different influence in the appraisal and coping of immediate stressors remain unexplored. Future research may investigate whether self-efficacy beliefs in specific domains, such as interpersonal relationships and health, might moderate the relationship between hassles and life satisfaction in late life.

The study exhibits strength in its measure of stressors and inclusion of covariates. The study minimized the risk of confounding measures of stressors and stress responses by focusing on the occurrence of stressors rather than individuals’ subjective evaluations of events (Dohrenwend et al., 1984). Specifically, the hassle measure was modified to eliminate the emphasis on “severity” and focus solely on the occurrence of stressors. Additionally, by controlling for various established correlates with life satisfaction and stressors, the results are more robust. Controlling for these covariates avoids potential confound and ensures the findings more accurately reflect the moderating effect of self-efficacy on the relationship between stressors and life satisfaction.

## Conclusion

This is the first study to show that general self-efficacy buffers the adverse impact of negative life events on life satisfaction in older adults. Moreover, the findings indicate that the extent to which self-efficacy moderates the relationships between stressors and life satisfaction in older adults varies depends on the types of stressors. The study contributes to theory by suggesting that self-efficacy purportedly facilitates the recovery of wellbeing after stressful life events, rather than the immediate appraisal and management of hassles. Strategies to strengthen self-efficacy can be incorporated into therapeutic interventions and community programs designed to maintain and enhance wellbeing and resilience in the older population.
